# DisoMCS: Accurately Predicting Protein Intrinsically Disordered Regions Using a Multi-Class Conservative Score Approach

**DOI:** 10.1371/journal.pone.0128334

**Published:** 2015-06-19

**Authors:** Zhiheng Wang, Qianqian Yang, Tonghua Li, Peisheng Cong

**Affiliations:** Department of Chemistry, Tongji University, Shanghai, China; University of Rome Tor Vergata, ITALY

## Abstract

The precise prediction of protein intrinsically disordered regions, which play a crucial role in biological procedures, is a necessary prerequisite to further the understanding of the principles and mechanisms of protein function. Here, we propose a novel predictor, DisoMCS, which is a more accurate predictor of protein intrinsically disordered regions. The DisoMCS bases on an original multi-class conservative score (MCS) obtained by sequence-order/disorder alignment. Initially, near-disorder regions are defined on fragments located at both the terminus of an ordered region connecting a disordered region. Then the multi-class conservative score is generated by sequence alignment against a known structure database and represented as order, near-disorder and disorder conservative scores. The MCS of each amino acid has three elements: order, near-disorder and disorder profiles. Finally, the MCS is exploited as features to identify disordered regions in sequences. DisoMCS utilizes a non-redundant data set as the training set, MCS and predicted secondary structure as features, and a conditional random field as the classification algorithm. In predicted near-disorder regions a residue is determined as an order or a disorder according to the optimized decision threshold. DisoMCS was evaluated by cross-validation, large-scale prediction, independent tests and CASP (Critical Assessment of Techniques for Protein Structure Prediction) tests. All results confirmed that DisoMCS was very competitive in terms of accuracy of prediction when compared with well-established publicly available disordered region predictors. It also indicated our approach was more accurate when a query has higher homologous with the knowledge database.

**Availability:**

The DisoMCS is available at http://cal.tongji.edu.cn/disorder/.

## Introduction

The intrinsically unstructured/disordered proteins (IUPs/IDPs) or intrinsically unstructured/disordered regions (IURs/IDRs) that do not possess stable secondary or tertiary structures play a crucial role in transcriptional regulation, translation and cellular signal transduction [1]. Even though these proteins lack intrinsic structure, they are able to bind to many different macromolecular partners when functioning in protein synthesis and protein interactions. Their prevalence is also associated with a number of human diseases [2], including cancer [3], cardiovascular disease [4], neurodegenerative diseases [5], genetic diseases [6], and amyloidosis [7, 8]. Therefore accurate prediction of disordered regions from a protein sequence is a key for the elaboration of the structural and functional hierarchy of proteins.

Predictions of disorder has a major role in directing laboratory experiments that are leading to the discovery of disordered proteins, and thereby leading to a positive feedback loop in the investigation of proteins. Importantly, the annotations of the IDPs are collected at a rather slow pace compared with the growing number of known, non-redundant protein sequences [9]. In recent decades, prediction of IDPs has attracted the attention of many researchers, and a number of prediction methods have been developed, and numerous characteristics of disordered regions are derived from protein sequences, such as low complexity [10], high net charge [11], and low content of hydrophobic amino acids [12]. At the same time, these motivate the development of computational models for the prediction of the disordered regions and have led to a growth in the number of IDR predictors.

The development of the widely adopted statistics and machine-learning methods of predicting IDPs and IDRs have been based on historical data for the last 30 years. The pioneer prediction for IDPs [13] was proposed by Williams in 1979 based on amino acid sequence. While a formal predictor was published by Obradovic and coworkers, they used three different algorithms, logistic regression, discriminant analysis, and an artificial neural network (ANN), named PONDR VL-XT to predict the disordered structures of proteins [14]. A slightly higher accuracy was given by ANN.

In the first decade of this century, many state-of-the-art predictors have been developed. A tool to identify regions of globularity and disorder within protein sequences, GlobPlot [15], was proposed. The regional order neural network (RONN) software [16] was published in 2005. Coeytaux presented the prediction of unfolded segments in a protein sequence based on amino acid composition [17]. Jaime P et al. presented a simple and versatile tool, Foldindex, for predicting intrinsically unfolded [18]. Tosatto’s group constructed Spritz for a disordered region [19] by support vector matching (SVM). A two-level SVM prediction system, POODLE-L, appeared in 2007 [20] for reliably predicting long disordered regions. DisPSSM [21] employed position-specific scoring matrices (PSSMP) with respect to physicochemical properties to identify the disordered regions of a query protein. DISOclust [22] predicted intrinsic disorder prediction from the analysis of multiple protein fold recognition models. A META-Disorder prediction method [23] molded various sources of information predominantly obtained from orthogonal prediction methods. CDF-all [24] based on various cumulative distribution functions to give a consensus prediction of intrinsically disordered proteins. During this period, there were numerous web servers available for users [18, 25–30] to predict IURs using their query sequences.

In modeling methodology, ANNs and SVMs were widely utilized techniques in the predictions of IDPs. Other predictions were exploited in this field, such as Spectral Graph Transducers (SGTs) [31], Bayesian methods [32], and Conditional Random Fields (CRFs) [29]. SGTs used the information from structure-unknown proteins in order to avoid training data sparseness. It predicted disordered structures with training on a huge amount of structure-unknown sequences as well as structure-known sequences [31]. The Bayesian classifier method [32] had been applied in the structure predictions of IDPs. Wang et al. [29] used CRFs to predict the intrinsic disorder in proteins. Compared to ANNs and SVMs, CRFs were able to take into account the interrelated information between two labels of neighboring residues. Recently, a number of IUR predictors have emerged, which usually had characteristics of large-scale prediction, higher accuracy prediction and unique model coverage of short and long disordered regions. PreDisorder [33] was proposed as an ab initio sequence-based predictor of protein disordered regions. MFDp [34]used two-layered architecture to predict IURs. MetaDisorder [35] was a meta-server for the prediction of IUPs. Tosatto’s group presented two new predictors, CSpritz [36] and ESpritz [37]. Zhou’s group demonstrated SPINE-D [38], which utilized new features and accurately predicted large-scale IUPs and IURs. Chen’s group presented DNdisorder [39] which used boosting and deep networks. These excellent predictors performed better in CASP (Critical Assessment of techniques for protein Structure Prediction) comparing with previous methods. Becker et al. also presented a predictor [40], which was competitive in terms of accuracy with respect to the state-of-the-art.

Here, we propose a novel predictor, DisoMCS, which is a more accurate predictor of protein intrinsically disordered regions. The DisoMCS bases on a new defined near-disorder region and an original multi-class conservative score (MCS) obtained by sequence-based structural similarity. DisoMCS utilizes a non-redundant data set as the training set, MCS and predicted secondary structure as features and conditional random field as the classification algorithm. In predicted near-disorder regions a residue is determined as order or disorder according to the optimized decision threshold. DisoMCS was used in cross-validation, large-scale prediction, independent tests and CASP tests.

## Materials and Methods

### Datasets

All data used in our approach were created from sequences and structures deposited at the PDB [41]. All the sequences comprising NMR structures and the X-ray crystallographic structures until December 31, 2011(containing 72,254 entries), with a length greater than 60 amino acids, were first collected. Disordered residues were defined as those missing backbone C-alpha atoms according to the definition of CASP [42]. Other definitions of disorder, such as order transform to disorder, population propensities [43], are hard to be used for performance comparison and are not adopted in this study. A database (referred as MCSbase) was obtained by using PISCES [44] with a sequence identity cut-off value of 99%. This returned 32,427 entries and their disorder/order structure elements were composed together. These sequences and structure elements were saved as a single file using FASTA format. Then a program (‘makeblastdb’ in BLAST+ toolkit) was carried out, and MCSbase was obtained. MCSbase is a knowledge database for multiple sequence analysis and BLAST-compatible database. Then, the similarity of pairwise sequences was cutoff at 25% sequence identity using PISCES. In total, this left 4,803 non-redundant protein chains (referred as DS4803). We randomly selected 3,803 chains (referred to as DS3803) from the DS4803 dataset as the training set to perform a cross validation test. DS3803 consisted of 925,291 residues, of which 43,837 (about 4.74%) were annotated as disordered residues. The remaining 1000 chains (referred to as DS1000) were used as an independent test set, in which there were 243,229 residues, 11,506 residues (about 4.73%) were disordered.

A benchmark dataset, widely used in the literature was defined as DS723 in this study. DS723 was compiled by Baldi and colleagues [45], and contained 723 entries, with 215,612 residues, of which 13,909 (about 6.50%) were defined as disordered. These protein sequences were also extracted from the PDB, with the following constraints: crystal structures with resolutions higher than 2.5 Å, greater than 30 amino acids in length, disordered regions of at least three residues in length and a sequence identity lower than 30%.

In addition, to examine the performance of our approach, another independent test dataset, named DS495, was constructed. The selection process of DS495 was similar to DS4803. First, all sequences released by the PDB between January 1, 2012 and June 30, 2012 were collected. Sequences greater than 60 amino acids in length were selected (containing 1,799 entries). These entries were combined with DS4803, and the similarity of pairwise sequences was cutoff at 25% sequence identity using PISCES. The test set contained 495 proteins with 132,062 residues, of which 7,889 (about 5.97%) were disordered residues. Moreover DS495 was divided into four regions according to their sequence identities to MCSbase for detail analysis.

The CASP10 data set was used to compare with several other methods. It was downloaded from the official website (URL: http://predictioncenter.org/casp10/). The most sequences of CASP10 targets have lower than 30% sequence identities with what have been stored in PDB before 2012.

Another independent testing set was DisProt. The DisProt was collected from DisProt database [46] (URL: http://www.disprot.org/). In order to avoid sequence similarity with MCSbase we selected the sequences that were in version 6.02 (Released at 2013-05-24) and not in version 6.01 (Released at 2012-10-15). There were ten sequences left. After removing three all disorder/order entries we reserved seven sequences, in which there were 12,029 residues, and 2645 residues were disordered (21.99%).

### Definitions of near-disorder region

**Fig 1:**
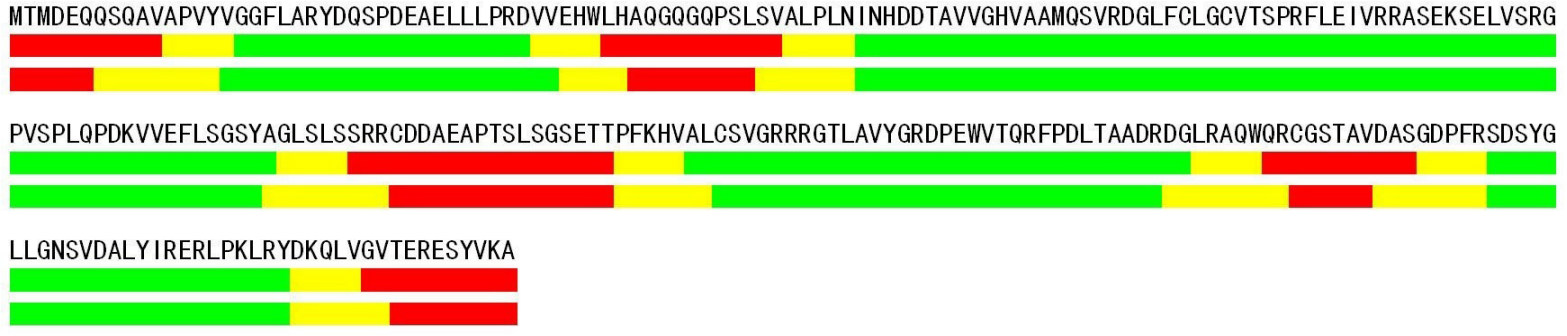
The top line represents a protein sequence (from PDB, ID:1CMV:A). The second line is the real definitions of ordered regions (green), disorder regions (red) and near-disorder regions (yellow, K = 5). The third line is the prediction result of our approach.

Recently, there has been a tendency to transform binary classification into a three-class problem. Zhou [38] defined ordered residues and disordered residues in short and long disordered regions in his predictor and reduced them into a two-state classification after prediction. Cheng [47] defined false boundary, near boundary and away boundary and constructed two predictors to identify protein domain boundaries. In this study, we define a near-disorder region in the start and the end terminus of an ordered region (Fig 1). A near-disorder contains K residues, which are the boundary regions of an ordered region. There are three benefits to the additional near-disorder region: (i) improvement of the imbalance between the positive and negative without pruning; (ii) analysis of prediction errors that often take place in these regions; and (iii) adjusting decision thresholds in these regions to improve performances.

### Multi-class Conservative Score

The innovative technology in this study is the multi-class conservative score approach. The MCS is generated by sequence-based structural similarity. For a query, sequence alignment is first performed against the MCSbase using PSI-BLAST [48] (with six iterations and 500 maximum sequences, other parameters are set as the default) to find homologous sequences relative to the query sequence. The matched piece-wise local sequences are then selected according to the e-values that are below a given threshold (for example 10). All the selected sequences are ranked according to their e-values in ascending order, and the top S (default is 10) of these sorted sequences that are considered as containing rich homologous information are reserved (if the number of the selected sequences were less than S, all the selected sequences would be retained). There are three elements for each amino acid position in the query sequences. For the matched sequences the order, near-disorder and disorder elements are counted in three boxes. These boxes then constitute an order/disorder profile of the original sequence. The profile is a probability and is defined as:

$$MSC_{s}^{p}=\sum_{L}A\left(p,s\right)/\sum_{S}\sum_{L}A\left(p,s\right)$$ (1)

Where L is the number of matched sequences. p is the position of the amino acid in the query sequence and *s* is one of three elements: order, disorder and near-disorder. *A*(*p*,*s*) is a binary (0, 1) value. When the state of the p amino acid in the matched sequences is s *A*(*p*,*s*) is 1, otherwise 0. In the denominator, the summation is carried out for these three states.

Finally, the MCS is exploited as a feature to identify the disordered region in the sequence. The MCS is a distinctive PSSM-like profile composed after alignments and has rich information about order, near-disorder and disorder.

### DisoMCS architecture

**Fig 2:**
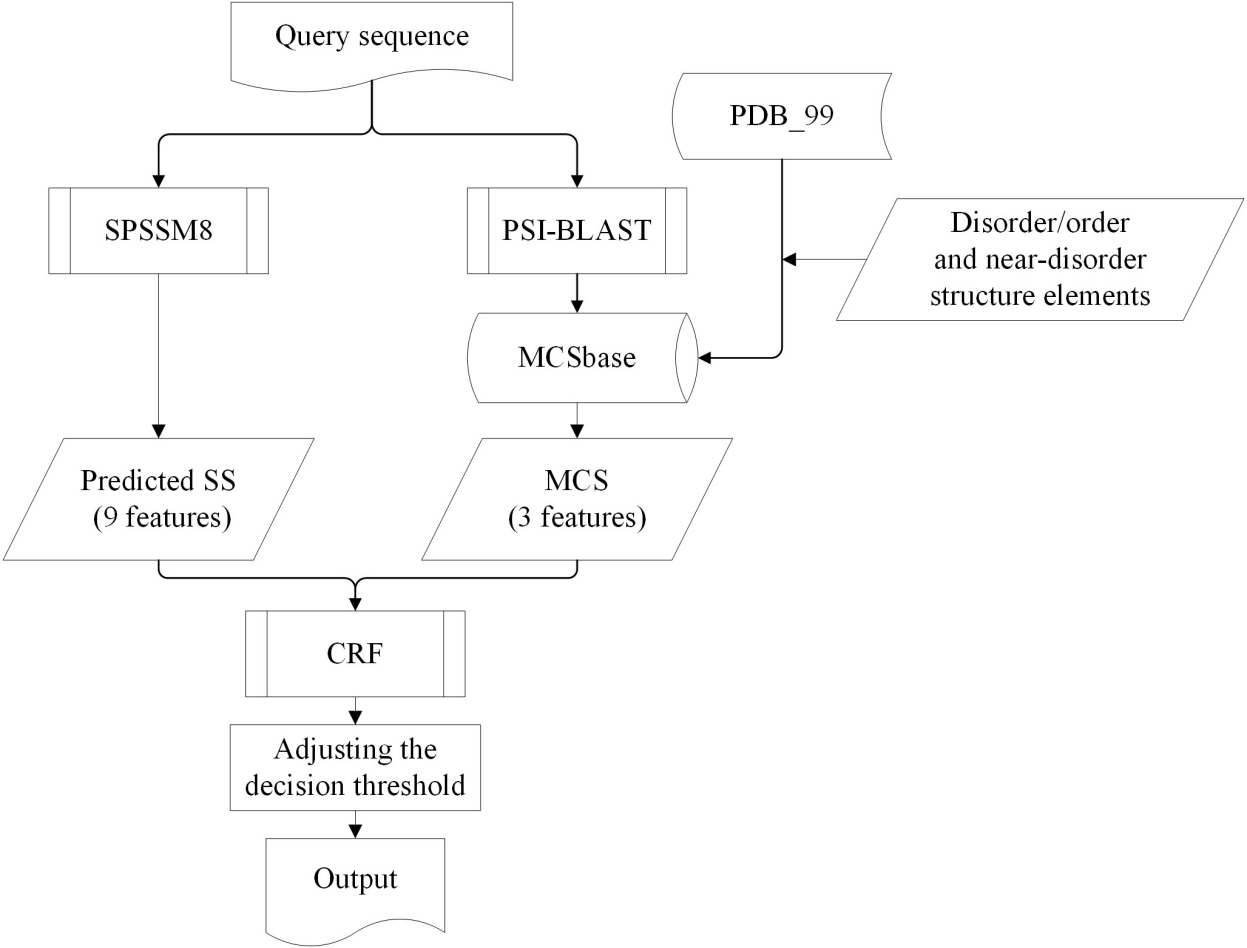
The flowchart of the DisoMCS. The DisoMCS used two kinds of features: MCSs and predicted secondary structures, giving a total of twelve features, and a conditional random field as the classification algorithm.

The architecture of DisoMCS is shown in Fig 2.

Only sequence is needed for prediction of IDRs. A query sequence is used to generate two kinds of features: MCSs and predicted secondary structures, giving a total of 12 features. There were three features of MCSs which were the profiles for order, near-disorder and disorder respectively. The MCS features are obtained by BLAST alignment against MCSbase, and are containing the homologous information of order, near-disorder and disorder regions. A MCS element is calculated according to the formula (1). There were nine features represented predicted secondary structures with orthogonal coding. The predicted secondary structure was obtained by using SPSSM8 [49] which could predict eight-state secondary structure of amino acids for a query sequence. The core of the SPSSM8 was a large database which contained 9 million sequences derived from the NR database (NCBI; as of 2009, 9,069,431 proteins were included) and putative structural information. There are nine elements of SPSSM8 output (eight-state secondary structure and a “-”). In total the 12 features were used as input features to CRF. When the queries are training sequences a CRF modelling routine is carried out to construct a model. When the queries are testing sequences we use a prediction routine to predict IDRs based on the obtained model.

Conditional random fields are powerful probabilistic frameworks to label and segment sequential data. As a discriminative model, CRFs do not need to model the visible observation sequence, and they directly model the conditional distribution, which results in the relaxation of strong independence assumptions over the observation sequence. Therefore, CRFs can achieve improved labeling and prediction performance. Moreover, CRFs are superior to many other machine learning methods in terms of speed without a slide window. In our approach, CRFs were utilized for modeling and prediction. We used the Unigram template for CRFs, the template that we generated considered four upward variables and four downward variables in a row, and then, all the variables in the column were traversed. We set all the parameters for modeling by default. We applied the CRF++0.54 [50], developed by TakuKudo, which is a simple, customizable, and open source implementation. The benefit of using CRF++0.54 is that it enables us to redefine the feature sets and specify the feature templates in a flexible way (CRF++0.54 is available at: http://crfpp.sourceforge.net/).

### Measuring performance

The performance of our predictions was assessed by multiple measures. For the binary predictions, we calculated sensitivity [Sn = TP/ (TP+FN)], specificity [Sp = TN/ (TN+FP)], accuracy [ACC = (Sens + Specificity)/ 2] and the Mathews correlation coefficient (MCC).

$$\text{MCC}=\frac{TP\times TN-FP\times FN}{\sqrt{\left(TP+FP\right)\left(TP+FN\right)\left(TN+FP\right)\left(TN+FN\right)}}$$ (2)

We also use the weighted score S_w_,

$$S_{w}=\frac{Wd\times TP-Wo\times FP+Wo\times TN-Wd\times FN}{Wd\times Nd+Wo\times No}$$ (3)

We observed there was a linear relationship between the weighted score and sensitivity and specificity (S_w_ = Sens +Spec–1) [51]. TP, TN, FN and FP are the number of true positives, true negatives, false negatives and false positives, respectively (positive is disorder, negative is order). N_o_ and N_d_ are the total numbers of ordered and disordered residues, respectively; W_o_ and W_d_ are the total percentages of disordered and ordered residues, respectively. In addition, we used the receiver operating characteristic (ROC) curve and area under the ROC curve (AUC), as a measure of the quality of the probabilities. The statistical significance of the evaluation scores was determined by bootstrapping: 80% of the targets were randomly selected 1000 times, and the standard error of the scores was calculated.

## Results and Discussion

### Validation of features

In order to validate features and select an optimal combination of features, 5-fold cross validation tests were performed on the DS723. The length of defined near-disorder was adopted as five (k = 5). The feature selection and combination were attempted, including the MCS, the position-specific scoring matrix (PSSM) using PSI-BLAST against NCBI non-redundant (nr) amino acid sequence databases [48] and predicted secondary structures (SS) by SPSSM8 [49]. We made different combinations of the variable to examine the joint effect. The assessment of the features and their combinations are summarized in Table 1. Note that because the MCS feature was derived from MCSbase, when the features were generated using BLAST against the MCSbase, any sequence which has an exact match in the DS723 was discarded from the MCSbase for fairness. The residue in the predicted near-disorder region was determined as order or disorder according to the decision threshold (see next section).

**Table 1:** Prediction results using different variables on DS723.

Variables	Sens	Spec	MCC	ACC	Sw	AUC
	Value ±SE	Value ±SE	Value ±SE	Value ±SE	Value ±SE	Value ±SE
**PSSM**	24.61 0.51	99.28 0.02	0.3945 0.0054	61.93 0.24	23.88 0.51	0.8220 0.0039
**MCS**	56.60 0.98	99.03 0.04	0.6554 0.0075	77.83 0.48	55.63 0.98	0.9339 0.0030
**SS**	51.09 0.83	98.41 0.12	0.5698 0.0103	74.77 0.42	49.50 0.85	0.8806 0.0040
**PSSM+SS**	51.87 0.82	98.47 0.10	0.5799 0.0092	75.12 0.43	50.34 0.82	0.8917 0.0034
**MCS+PSSM**	60.85 0.85	98.76 0.04	0.6666 0.0067	79.80 0.41	59.61 0.86	0.9353 0.0029
**MCS+SS**	65.11 0.82	98.86 0.04	0.7039 0.0063	81.97 0.45	63.98 0.82	0.9432 0.0026
**MCS+PSSM +SS**	65.18 0.82	98.80 0.04	0.6996 0.0062	82.01 0.43	63.97 0.82	0.9466 0.0028

PSSM is a widely used feature for prediction the protein intrinsically disordered region. However, it did not perform well in our experiments: Sw was only 23.88%. SS feature performed better. Sw achieved 49.50%. When MCS was used as a feature, Sw achieved 55.63%. It was obvious that MCS was the extraordinary useful feature for IDR prediction. When MCS and SS were used as features, Sn and Sp was 65.11% and 98.86% respectively, and Sw achieved 63.98%. The improvement of the corresponding areas under the curve (AUC) highlighted the effect of MCS. The AUC value, after using MCS and SS was 0.9432 (S1 Table). When the PSSM was added again, the performance was almost the same. So we used MCS and SS as features in nest studies.

### Adjusting the decision threshold

DS723 was used to demonstrate the behavior of the new defined near-disorder region. First, due to the definition of the near-disorder region, the imbalance problem was improved, the ratio of the number of the ordered residues to the number of disorder residues is changed from 14.5:1 to 6.9:1:0.6, which is the ratio of residue numbers (order: near-disorder: disorder). Thus the two-class problem is transformed to three-class problem.

**Fig 3:**
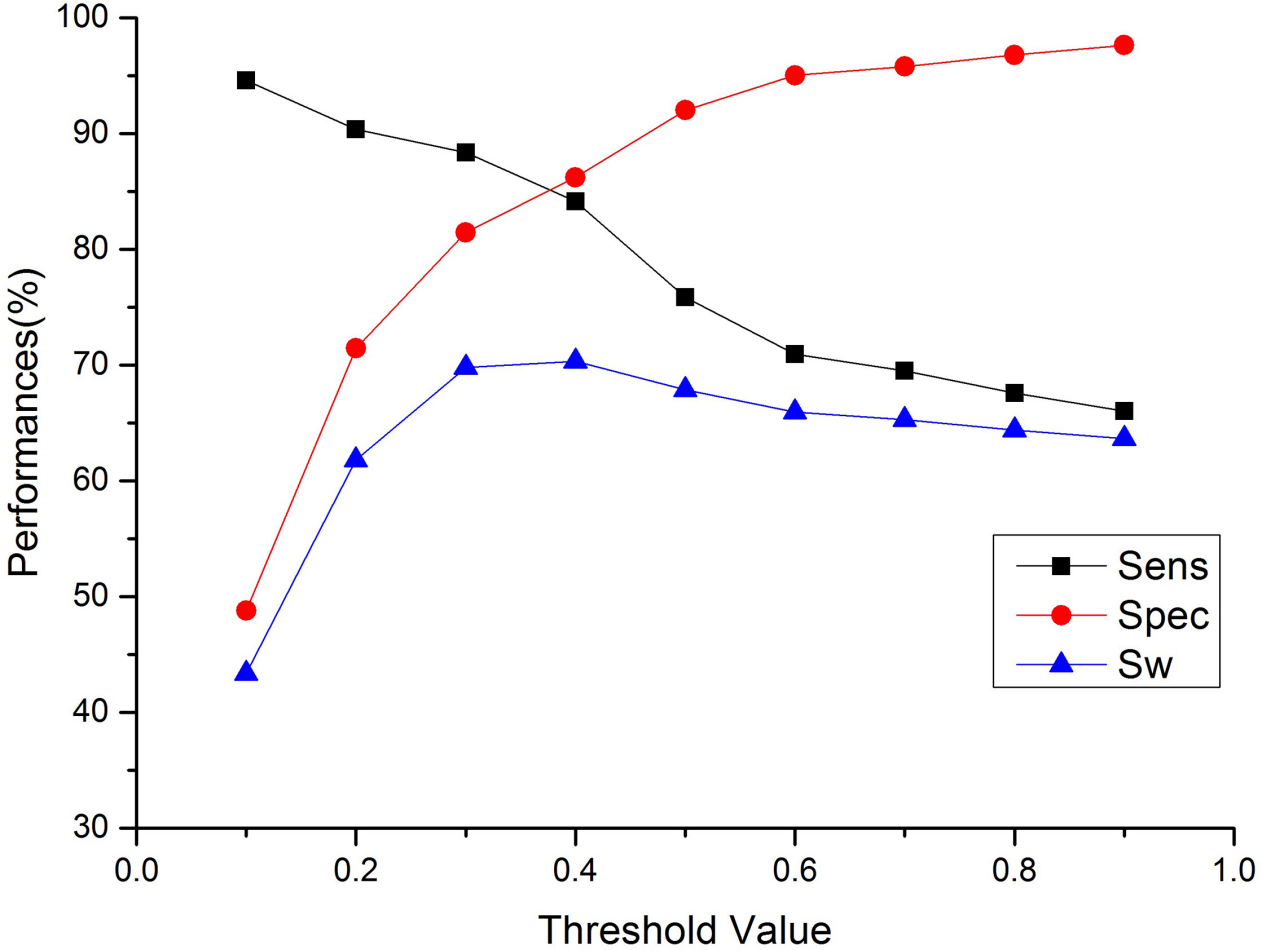
Adjusting the decision threshold of predicted near-disorder regions. The Sw achieves maximum 70.33%when the decision threshold is 0.4.

Second, it is well-known that the predicted errors often take place in the terminus of predicted ordered regions, in which a residue is a disorder residue but is predicted as an order residue. The addition of near-disorder regions cover these regions (Fig 1) and reduce mistakes. We determine the residues in the predicted near-disorder regions to be either order or disorder according to a decision threshold. If the near-disorder probability of a residue is greater than a given threshold when this residue is not determined as a disorder residue, the residue would be determined as disorder, otherwise the residue would belong to order. The performances of adjusting the decision thresholds are shown in Fig 3. When the decision threshold is 0.4, we achieve the maximum 70.33% of Sw, which is used as the criterion of optimization of the decision threshold. The decision threshold of the near-disorder region of 0.4 is referred to as scheme I in the following text. In Fig 3, we also show the effects of the decision threshold when it is not dominant, indicating the adjustment of the decision threshold could greatly change the sensitivity and specificity.

**Fig 4:**
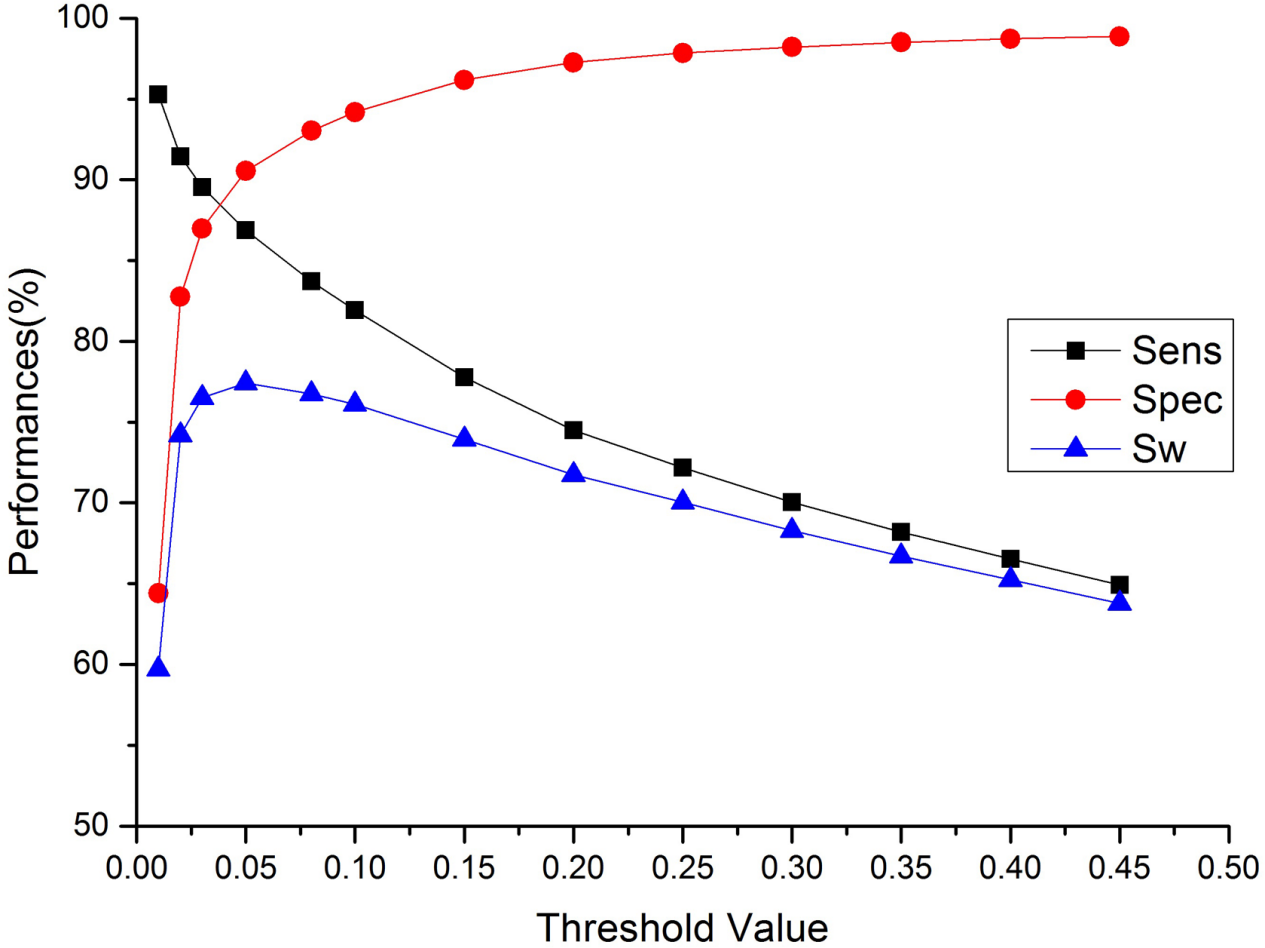
Adjusting the decision threshold of predicted disorder. The Sw achieves maximum 76.55% when the decision threshold is 0.03.

Third, another feasible adjustment of the decision threshold is focused on the disordered regions. It is also possible to adjust the decision threshold of the disorder probability to balance the sensitivity and specificity (Fig 4) as previously performed [37, 38]. If the probability of a residue is greater than a given threshold, the residue would be determined as a disorder, otherwise the residue would belong to an order. The Sw score achieves maximum 76.55% when the decision threshold is 0.03, and this is called scheme II in the following text.

When the decision threshold is 0.4 according scheme I Sw achieves 70.33%, and Sn and Sp is 76.47% and 93.86% respectively (Table 2). When the decision threshold is 0.03 according scheme II Sw achieves 76.55%, and Sn and Sp is 89.55% and 87.00% respectively. According to our experiments scheme I could not have a great effect on Sw, however, it does not decrease Sp evidently. Scheme II has the ability to greatly improve Sn. When the testing is similar with the training, Sw will achieve or approximate to the maximum (S2 Table).

**Table 2:** The performances comparison with various adjustments on the DS723 dataset.

	Sens	Spec	Sw
	Value ±SE	Value ±SE	Value ±SE
**Scheme I**	76.47 0.82	93.86 0.04	70.33 0.82
**Scheme II**	89.55 0.55	87.00 0.32	76.55 0.61

In summarizing the multi-class conservative score approach proposed in this study there are three issues of the approach: (i) definition; (ii) MCS feature; and (iii) adjusting the decision threshold. The multi-class conservative score approach has been confirmed to benefit the identification of protein disordered regions. Similar to other alignment-based methods the performance of MCS would lose its effect if there is no homology information obtained when the alignment is carried out. Therefore, we believe as more structures of proteins are determined it will be easier to obtain information of homological alignment.

### Ensemble of protein disorder prediction

We utilized our approach on ensemble of protein disorder, to allow for large-scale prediction. We first performed 5-fold cross validation on DS3803. We then trained using DS3803 and predicted the independent test set of DS1000. The used features were MCS and SS, and the decision threshold was 0.4 according to scheme I or 0.03 according to scheme II, which were obtained by optimizing based on DS723. When we used BLAST against MCSbase, any sequence which had an exact match in the DS3803 and DS1000 was discarded from the MCSbase. The 5-fold cross-validation is a relatively strict cross-validation method used to estimate how accurately a predictive model will perform in practice, and is important in guarding against testing hypotheses suggested by the data.

The accurate performances confirmed using our predictor was good even in the case of large-scale validation. For DS1000, when we adjusted the threshold using scheme I (0.4), the Sn was 82.60%, and Sw was 74.40%. When we adjusted the threshold with scheme II (0.03), we achieved a Sn of 90.20% and a Sw of 78.64%. However for Sp we achieved 91.80% and 88.52% with scheme I and scheme II respectively. It was obvious that the adjusted threshold with scheme II was the extraordinary useful feature for IDR prediction. Table 3 also shows that the AUC value achieved 0.9534 of DS3803 and 0.9577 of DS1000. These demonstrated our approach had strong ability of predicting protein intrinsically disordered regions (S3 Table).

**Table 3:** Prediction results of 5-fold cross-validation and independent test sets.

	Sens	Spec	MCC	ACC	Sw	AUC
	Value ±SE	Value ±SE	Value ±SE	Value ±SE	Value ±SE	Value ±SE
**DS3803** ^**a**^	84.97 0.40	88.26 0.02	0.4396 0.0044	86.62 0.06	73.24 0.40	0.9524 0.0012
**DS1000** ^**a**^	82.45 0.82	88.34 0.04	0.4336 0.0062	85.40 0.09	70.79 0.92	0.9577 0.0023
**DS3803** ^**b**^	89.06 0.27	88.33 0.12	0.4562 0.0027	88.69 0.14	77.39 0.29	0.9524 0.0012
**Dd1000** ^**b**^	90.20 0.52	88.44 0.23	0.4638 0.0056	89.31 0.30	78.64 0.59	0.9577 0.0023

a: scheme I (0.4).

b: scheme II (0.03).

### Independent test and comparison to other methods

To examine the performance of our approach we trained on the DS4803 and predicted the independent test set of DS495. The used features were MCS and SS, and the decision threshold was 0.03 according to scheme II. We designed DS495 as a set of proteins with low similarity to proteins in the DS4803 set to evaluate the approach. Combining DS495 with DS4803, the similarity of pairs of sequences was cutoff at 25% sequence identity. Moreover DS495 was divided into four regions according to their sequence identities to MCSbase. Table 4 shows that the performances of the proposed method and other methods (Disopred [30], ESpritz [36] and IUPred [25]) in different regions. Except [0,15] region, we achieved the maximum values on Sn, Sw and ACC. Even for very low sequence identity region (<15%), the Sn was 76.03%, which was better than others. The Sp was 81.88%. This result indicates that the proposed prediction model has greater discrimination for disordered regions (S4 Table). It also indicates our approach is more appropriate for queries that could find homologous in the knowledge database.

**Table 4:** Performance comparison with various methods on the independent dataset.

	Identity*	Sens	Spec	Sw	ACC	MCC
**DisoMCS**	**[0,15]%**	**76.03**	81.88	57.91	78.96	0.3611
	**[15,30]%**	**78.89**	90.32	**69.21**	**84.60**	0.4424
	**[30,60]%**	**81.42**	91.91	**73.33**	**86.66**	0.4987
	**[60,90]%**	**87.63**	89.43	**77.06**	**88.53**	0.5146
**Disopred**	**[0,15]%**	63.36	**98.15**	**61.51**	**80.75**	**0.6566**
	**[15,30]%**	56.24	**99.12**	55.36	77.68	**0.6422**
	**[30,60]%**	54.83	**99.07**	53.90	76.95	**0.6297**
	**[60,90]%**	73.49	**99.06**	72.55	86.27	**0.7712**
**ESpritz**	**[0,15]%**	74.96	86.16	61.12	80.56	0.4106
	**[15,30]%**	72.87	90.00	62.87	81.43	0.4016
	**[30,60]%**	75.99	89.68	65.66	82.83	0.4197
	**[60,90]%**	88.12	87.73	75.85	87.92	0.4864
**IUPred**	**[0,15]%**	46.10	90.55	36.66	68.33	0.2917
	**[15,30]%**	32.37	95.34	27.71	63.86	0.2510
	**[30,60]%**	40.59	95.32	35.92	67.96	0.3198
	**[60,90]%**	53.33	90.68	44.01	72.01	0.3254

*: sequence identity hit to MCSbase

The sequences in DisProt set are rich disordered sequences. The decision threshold was 0.03 according to scheme II. The ACC was 74.18%. The Sw achieved 48.36%, and Sn and Sp was 64.84% and 83.53% respectively. In DisProt there was a query whose sequence identity with MCSbase was very low. It indicates that if very low sequence identity entry is concerned ab initio is especially required in disorder prediction because highly disordered sequences and disordered proteins should be absent from the PDB. In that case, machine learning approach would not be competitiveness with ab initio.

### Comparison on CASP10 data

**Fig 5:**
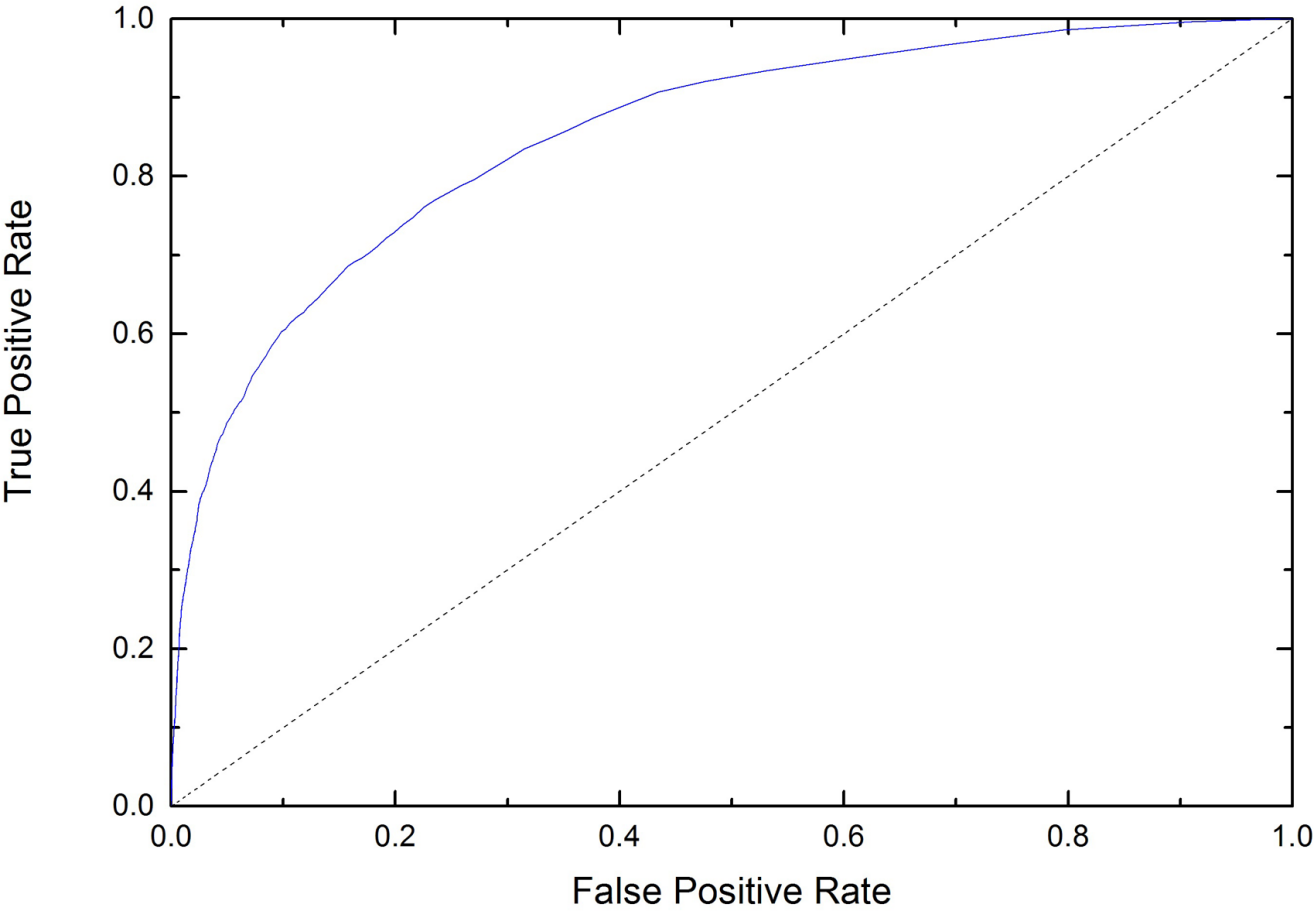
ROC plot of CASP10.

In order to compare our approach to the state-of-the-art methods, we used the data from the recent CASP10 experiment [42]. Table 5 shows the results for 94 CASP10 queries. CASP10 was a difficult blind test, as no previous information was available. We used DS4803 as a training set, with MCS and predicted secondary structure as an input, and the scheme II (0.03) to adjust the threshold in the predicted near-disorder. When we used BLAST against MCSbase, we couldn’t find any sequence which had an exact match in the CASP10. The performances of our approach and recently reported state-of-art approaches [39] are listed in Table 5 (S5 Table). Comparing with other methods, our approach achieved the maximum of ACC and Sw. DisoMCS was more accurate than all the reported approaches. We achieved an ACC of 77.83%, a Sw of 55.66%. The Roc of CASP10 is showed in Fig 5.

**Table 5:** Performance on the CASP10 dataset.

Predictor	ACC		Sensitivity		Specificity		Sw		AUC	
	Value	±SE	Value	±SE	Value	±SE	Value	±SE	Value	±SE
**DisoMCS**	77.83	0.81	72.66	1.5	83.00	0.95	55.66	1.7	0.8526	0.008
**metaprdos2**	77.06	0.92	64.73	1.4	89.40	0.98	54.12	1.8	0.8727	0.006
**PreDisorder**	76.86	0.67	67.19	1.7	86.34	0.94	53.73	1.3	0.8394	0.006
**POODLE**	76.84	0.78	62.74	1.6	90.94	0.26	53.68	1.6	0.8663	0.006
**PreDNdisorder**	76.55	0.75	61.74	1.8	91.36	0.61	53.10	1.5	0.8642	0.006
**ZHOU-SPARKS-X**	75.68	0.76	64.81	1.4	86.55	0.96	51.36	1.5	0.8588	0.006
**Dndisorder**	75.19	0.71	61.92	1.4	88.46	0.29	50.39	1.4	0.8480	0.006
**Cspritz**	75.13	1.40	66.31	1.3	83.94	2.40	50.25	2.9	0.8215	0.007
**Espritz**	73.16	1.60	59.24	1.4	87.08	2.60	46.31	3.2	0.8457	0.006
**espritz_nopsi_X**	71.98	0.97	53.10	1.5	90.87	0.77	43.97	2.0	0.8145	0.007
**PrDOS-CNF**	70.35	0.88	41.95	1.8	98.74	0.14	40.70	1.8	0.8956	0.005
**biomine_dr_mixed**	69.17	0.68	39.95	1.4	98.40	0.11	38.34	1.4	0.8844	0.006
**biomine_dr_pdb_c**	67.81	1.20	36.88	2.6	98.74	0.15	35.62	2.5	0.8815	0.006
**iupred_short**	63.26	0.70	30.68	1.5	95.84	0.25	26.52	1.4	0.6642	0.007

Note: the performances of the state-of-art were reported on [39]

In order to detail comparison, the performances of DisoMCS, Disopred [30], ESpritz [36] and IUPred [25] on different regions (FM (11 entries, other (4 entries), TBM (73 entries) and TBM-hard (6 entries)) of CASP10 are given in Table 6. For Sn, Sw and ACC measurements our approach is the best on all regions. These demonstrate that our approach is competitiveness with the state-of-art approaches.

**Table 6:** Performances comparison with various methods on different CASP10 regions.

		Sens	Spec	Sw	ACC	MCC
**DisoMCS**	FM	**70.20**	70.66	**40.86**	**70.43**	0.1933
	other	**97.73**	78.35	**76.07**	**88.04**	0.2931
	TBM	**72.37**	87.27	**59.64**	**79.82**	**0.4140**
	TBM-hard	**70.83**	69.64	**40.48**	**70.24**	0.1792
**Disopred**	FM	32.83	**98.05**	30.88	65.44	**0.3682**
	other	47.73	95.09	42.81	71.41	0.2934
	TBM	40.52	**96.55**	37.07	68.54	0.4026
	TBM-hard	37.50	95.44	32.94	66.47	0.2861
**ESpritz**	FM	33.33	93.25	26.58	63.29	0.2138
	other	84.09	90.69	74.78	87.39	**0.3862**
	TBM	56.30	88.40	44.70	72.35	0.3283
	TBM-hard	48.61	88.79	37.41	68.70	0.2310
**IUPred**	FM	0.00	94.24	-	47.12	-
	other	47.73	**95.80**	43.53	71.76	0.3156
	TBM	17.70	95.77	13.47	56.74	0.1583
	TBM-hard	25.00	**99.93**	24.93	62.47	**0.4772**

An example is a susd homolog (BACOVA_04803) from Bacteroides ovatus ATCC 8483 (CASP10 target T0664, PDB ID 4f53). It has 540 residues and 42 disordered residues (7.78%).

**Fig 6:**
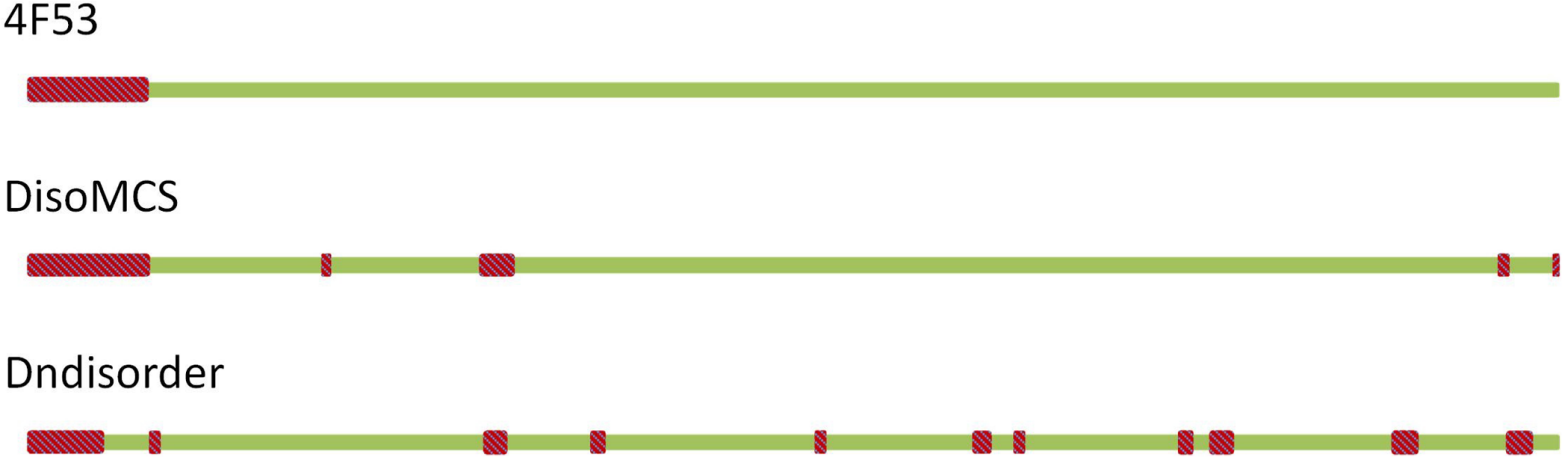
An example of CASP10.

We achieved a Sn of 100%, and a Sp of 94.8%. The false positive predictions were four short fragments (Fig 6). The Sn and Sp was 28.6% and 86.3% respectively for Dndisorder [38].

### Web servers

The DisoMCS server is available at http://cal.tongji.edu.cn/disorder/ for users to predict protein intrinsically disordered regions for query sequence(s). Users can press the "bookmark this page" add the URL link to their favorites through the browser menu, and they can use this link to retrieve the results at a later time.

## Conclusions

In this work, we have proposed DisoMCS for accurately predicting the disordered regions in proteins. It based on a multi-class conservative score strategy which was sequence-based structural similarity. DisoMCS was performed using a 5-fold cross-validation, large-scale prediction, independent test and CASP10 tests. The results demonstrated that DisoMCS was very competitive in terms of accuracy to well-established publicly available disordered region predictors. The core technology is an original multi-class conservative score which is based on a new defined near-disorder region, an effective MCS feature and adjustment of the decision threshold. The most distinct character of DisoMCS is that only two kinds of features are utilized during modeling and prediction. A small number of features and high accuracy allow our approach to compete with the state-of-the-art predictors of disorder regions. DisoMCS is more accurate when a query has high homologous with MCSbase. We believe that DisoMCS offers an accurate and efficient way to address many biologically relevant problems encountered with disordered proteins.

## Supporting Information

S1 Table Prediction results using different variables on DS723. (DOC)

S2 Table The performances comparison with various adjustments on the DS723 dataset. (DOC)

S3 Table Prediction results of 5-fold cross-validation and independent test sets. (DOC)

S4 Table Performance comparison with various methods on the independent dataset. (DOC)

S5 Table Performance on the CASP10 dataset. (DOC)
