# Supplementary material for: DisoMCS: Accurately Predicting Protein Intrinsically Disordered Regions Using a Multi-Class Conservative Score Approach
Source: PLoS One. 2015 Jun 19;10(6):e0128334. doi: 10.1371/journal.pone.0128334 (PMC4474717; doi:10.1371/journal.pone.0128334)
Supplement: S1 Table — (DOC) [file pone.0128334.s001.doc]

**Supplementary data**

TP, TN, FN and FP are the number of true positives, true negatives, false negatives and false positives, respectively (positive is disorder, negative is order).

**Table S1**. Prediction results using different variables on DS723

| **Variables** | **TP** | **FP** | **TN** | **FN** |
| --- | --- | --- | --- | --- |
| **PSSM** | 3418 | 1454 | 200249 | 10491 |
| **MCS** | 7874 | 1947 | 199756 | 6035 |
| **SS** | 7109 | 3224 | 198479 | 6800 |
| **PSSM+SS** | 7208 | 3088 | 198615 | 6701 |
| **MCS+PSSM** | 8459 | 2504 | 199199 | 5450 |
| **MCS+SS** | 9049 | 2294 | 199409 | 4860 |
| **MCS+PSSM +SS** | 9069 | 2428 | 199275 | 4840 |
