# Supplementary material for: DisoMCS: Accurately Predicting Protein Intrinsically Disordered Regions Using a Multi-Class Conservative Score Approach
Source: PLoS One. 2015 Jun 19;10(6):e0128334. doi: 10.1371/journal.pone.0128334 (PMC4474717; doi:10.1371/journal.pone.0128334)
Supplement: S2 Table — (DOC) [file pone.0128334.s002.doc]

**Supplementary data**

TP, TN, FN and FP are the number of true positives, true negatives, false negatives and false positives, respectively (positive is disorder, negative is order).

**Table S2**. The performances comparison with various adjustments on the DS723 dataset

|  | **TP** | **FP** | **TN** | **FN** |
| --- | --- | --- | --- | --- |
| **Scheme I** | 11240 | 12336 | 188577 | 3459 |
| **Scheme II** | 12451 | 26238 | 175465 | 1458 |
