# Supplementary material for: DisoMCS: Accurately Predicting Protein Intrinsically Disordered Regions Using a Multi-Class Conservative Score Approach
Source: PLoS One. 2015 Jun 19;10(6):e0128334. doi: 10.1371/journal.pone.0128334 (PMC4474717; doi:10.1371/journal.pone.0128334)
Supplement: S3 Table — (DOC) [file pone.0128334.s003.doc]

**Supplementary data**

TP, TN, FN and FP are the number of true positives, true negatives, false negatives and false positives, respectively (positive is disorder, negative is order).

**Table S3**. Prediction results of 5-fold cross-validation and independent test sets

|  | **TP** | **FP** | **TN** | **FN** |
| --- | --- | --- | --- | --- |
| **DS3803^a^** | 38685 | 103263 | 776504 | 6839 |
| **DS1000^a^** | 10266 | 26902 | 203876 | 2185 |
| **DS3803^b^** | 39038 | 102910 | 778544 | 4799 |
| **Dd1000^b^** | 10378 | 26790 | 204933 | 1128 |

a : scheme I (0.4).

b: scheme II (0.03).
