# Supplementary material for: DisoMCS: Accurately Predicting Protein Intrinsically Disordered Regions Using a Multi-Class Conservative Score Approach
Source: PLoS One. 2015 Jun 19;10(6):e0128334. doi: 10.1371/journal.pone.0128334 (PMC4474717; doi:10.1371/journal.pone.0128334)
Supplement: S4 Table — (DOC) [file pone.0128334.s004.doc]

**Supplementary data**

TP, TN, FN and FP are the number of true positives, true negatives, false negatives and false positives, respectively (positive is disorder, negative is order).

**Table S4**. Performance comparison with various methods on the independent dataset

|  | **TP** | **FP** | **TN** | **FN** |
| --- | --- | --- | --- | --- |
| **[0,15]%** | 2693 | 8187 | 37006 | 849 |
| **[15,30]%** | 1233 | 2952 | 27553 | 330 |
| **[30,60]%** | 1424 | 2647 | 30078 | 325 |
| **[60,90]%** | 907 | 1665 | 14085 | 128 |
