# Supplementary material for: DisoMCS: Accurately Predicting Protein Intrinsically Disordered Regions Using a Multi-Class Conservative Score Approach
Source: PLoS One. 2015 Jun 19;10(6):e0128334. doi: 10.1371/journal.pone.0128334 (PMC4474717; doi:10.1371/journal.pone.0128334)
Supplement: S5 Table — (DOC) [file pone.0128334.s005.doc]

**Supplementary data**

TP, TN, FN and FP are the number of true positives, true negatives, false negatives and false positives, respectively (positive is disorder, negative is order).

**Table** **S5**. Performance on the CASP10 dataset

|  | **TP** | **FP** | **TN** | **FN** |
| --- | --- | --- | --- | --- |
| **CASP10** | 1210 | 4041 | 19665 | 454 |
